# Extensive Hair Shaft Growth after Mouse Whisker Follicle Isolation, Cryopreservation and Transplantation in Nude Mice

**DOI:** 10.1371/journal.pone.0145997

**Published:** 2015-12-30

**Authors:** Wenluo Cao, Lingna Li, Benjamin Tran, Satoshi Kajiura, Yasuyuki Amoh, Fang Liu, Robert M. Hoffman

**Affiliations:** 1 AntiCancer Inc., San Diego, CA, 92111, United States of America; 2 Department of Surgery, University of California San Diego, San Diego, CA, 92103, United States of America; 3 Department of Anatomy, Second Military Medical University, Shanghai, 200433, China; 4 Department of Dermatology, Kitasato University, Sagimahara, 228–8555, Japan; National Cancer Institute, UNITED STATES

## Abstract

We previously demonstrated that whole hair follicles could be cryopreserved to maintain their stem-cells differentation potential. In the present study, we demonstrated that cryopreserved mouse whisker hair follicles maintain their hair growth potential. DMSO better cryopreserved mouse whisker follicles compared to glycerol. Cryopreserved hair follicles also maintained the hair follicle-associated-pluripotent (HAP) stem cells, evidenced by P75^NTR^ expression. Subcutaneous transplantation of DMSO-cryopreserved hair follicles in nude mice resulted in extensive hair fiber growth over 8 weeks, indicating the functional recovery of hair shaft growth of cryopreserved hair follicles.

## Introduction

Hair follicle stem cells expressing nestin were discovered in transgenic mice expressing nestin-driven green fluorescent protein (ND-GFP) [1]. The ND-GFP cells are located above the bulge area (BA) of the hair follicle, below the sebaceous gland. We have termed these cells hair-follicle-pluripotent (HAP) stem cells [1, 2].

HAP stem cells can differentiate into neurons, glial cells, smooth muscle cells, keratinocytes, and other cell types [3].

HAP stem cells transplanted to the severed sciatic nerve of the mousepromoted axonal growth of preexisting neurons and functional recovery [4]. Transplantation of HAP stem cells also promoted spinal cord repair and functional recovery [5].

HAP stem cells produced the whisker sensory nerve [6], when whisker follicles were placed in 3-dimensional Gelfoam histoculture, suggesting that a major function of the HAP stem cells is for growth of the follicle sensory nerve [6].

We have recently demonstrated that HAP stem cells differentiated to beating cardiac muscle cells [7].

We previously demonstrated efficient cryopreservation methods of the hair follicle which maintained the pluripotency of HAP stem cells. Whole mouse whisker follicles from GFP transgenic mice were cryopreserved by slow-rate cooling and storage in liquid nitrogen. Upon thawing and culture, the upper part of the hair follicle produced almost as many pluripotent hair spheres as fresh follicles [8].

In the present study, we demonstrate that isolated intact mouse whisker hair follicles could be cryopreserved such that they maintained their hair-shaft growth potential after transplantation to nude mice.

## Methods

### Ethics Statement

All animal studies were conducted with an AntiCancer Institutional Animal Care and Use Committee (IACUC)-protocol specifically approved for this study and in accordance with the principals and procedures outlined in the National Institute of Health Guide for the Care and Use of Animals under Assurance Number A3873-1. In order to minimize any suffering of the animals, anesthesia and analgesics were used for all surgical experiments. Animals were anesthetized by intramuscular injection of a 0.02 ml solution of 20 mg/kg ketamine, 15.2 mg/kg xylazine, and 0.48 mg/kg acepromazine maleate. The response of animals during surgery was monitored to ensure adequate depth of anesthesia. Ibuprofen (7.5 mg/kg orally in drinking water every 24 hours for 7 days post-surgery) was used in order to provide analgesia post-operatively in the surgically-treated animals. The animals were observed on a daily basis and humanely sacrificed by CO_2_ inhalation when they met the following humane endpoint criteria: prostration, skin lesions, significant body weight loss, difficulty breathing, epistaxis, rotational motion and body temperature drop. The use of animals was necessary to determine the hair growth potential of transplanted cryopreserved hair follicles. Animals were housed with no more than 5 per cage. Animals were housed in a barrier facility on a high efficiency particulate arrestance (HEPA)-filtered rack under standard conditions of 12-hour light/dark cycles. The animals were fed an autoclaved laboratory rodent diet (S1 ARRIVE Checklist).

### Animals

Transgenic mice with ND-driven GFP [1, 9] and non-transgenic (*nu/nu*) nude mice (AntiCancer, Inc., San Diego, CA), were used to this study according to the Ethics Statement described above.

### Isolation and Cryopreservation of Whisker Hair Follicles

**Fig 1:**
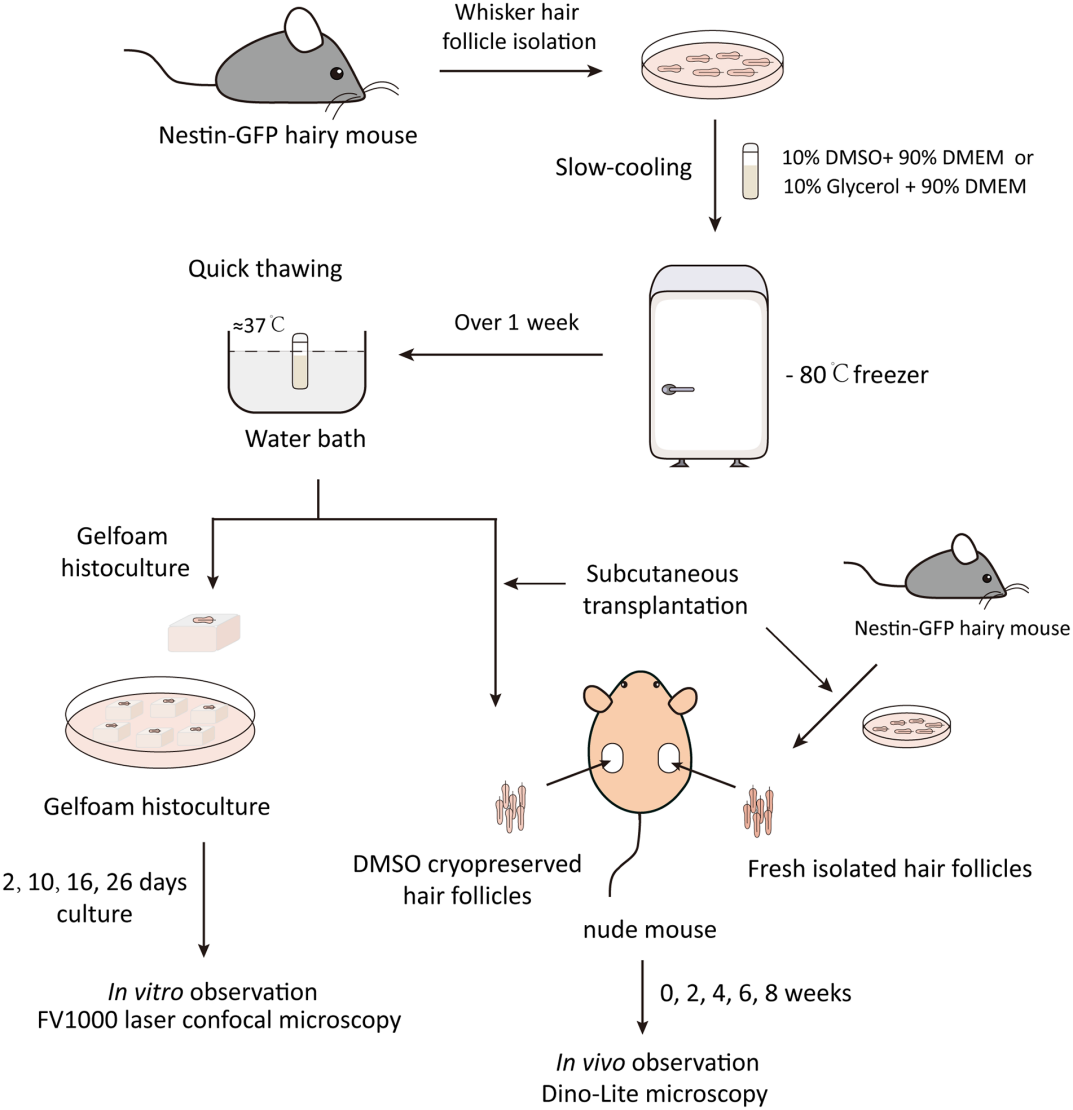
Schema of hair follicle isolation and cryopreservation, Gelfoam histocultured and transplantation.

Whicker hair follicles were carefully isolated by microdissection from nestin driven (ND)-GFP mice. The intact hair follicle bulb with the attached sensory nerve was gently removed from subctuaneous fat under a stereo dissecting microscope [6, 10, 11]. In order to cryopreserve the hair follicle, fresh isolated whole whisker hair follicles were then transferred to cryovials. Either 10% glycerol or DMSO with 90% FBS was added (1 ml) as the freezing medium (Fig 1). For short time period, cryovials containing hair follicles were stored in an -80°C freezer.

### Gelfoam^®^ Histoculture of Whisker Hair Follicles

After one week, the cryopreserved hair follicles were thawed at 37°C in a water bath with gentle shaking (Fig 1). The hair follicles were washed at DMEM medium for in vitro culture and in vivo transplantation. The cryopreserved hair follicles or fresh isolated hair follicles were carefully placed on sterile Gelfoam^®^ (Pharmacia and Upjohn Co., Kalamazoo, MI) hydrated. DMEM-F12 medium (GIBCO, Life Technologies, Grand Island, NY) containing B-27 (2.5%) (GIBCO), N2 (1%) (GIBCO) and 1% penicillin and streptomycin (GIBCO). The Gelfoam^®^ follicles cultures [6, 10, 11] were incubated at 37°C, 5% CO_2_ incubator. The medium was changed every other day. High-resolution images of hair follicle growth on Gelfoam^®^ were obtained with a confocal laser-scanning microscope (Fluoview FV1000, Olympus, Tokyo, Japan) [6, 10, 11].

### Immuohistochemistry of Hair Follicles

Hair follicles were fixed in 4% paraformaldehyde overnight at 4°C, after dehydration in 15% and 30% sucrose overnight at 4°C successively. Tissues were then embedded in the OTC freezing compound (Thermo Scientific, Kalamazoo, MI) and frozen sections of 10 μm were prepared with a CM1850 cryostat (Leica, Buffalo Grove, IL). The frozen sections were washed with PBS three times. After incubation with 0.4% Triton X-100 for 10 minutes, the sections were incubated with 20% BSA in PBS containing 0.4% Triton X-100 for 1h at room temperature (RT). Primary antibody, anti- p75^NTR^ (rabbit, 1:400, cell Signaling), was applied at 4°C overnight. After three washes in PBS, secondary antibody, Alexa Fluor^®^ 555-conjugated anti-rabbit (1:100, Jackson), was applied in PBS at RT for 1 h. After three washes in PBS, the nuclei were stained with DAPI (1:2000, Invitrogen) for 5 min. Images were obtained with a confocal FV1000 laser-scanning microscopy.

### Subcutaneous Transplantation of Hair Follicles

Non-transgenic nude mice were anesthetized with the ketamine mixture (described above).Thawed frozen hair follicles were transplanted into the subcutis on the right flank of nude mice and fresh isolated hair follicles were transplanted to the left flank as controls. A skin flap was made to expose the growing hair follicle every 2 weeks (2, 4, 6, and 8 weeks) in order to observe hair growth. Images were obtained with the Dino-Lite microscope portable imager (AM4113TGFBW Dino-Lite Premier; AnMo Electronics Corporation, Taiwan) [12].

### Measurement of Hair Shaft Length

The in vivo hair follicle images obtained with the Dino-Lite were used to determine the length of each hair shaft using Image Pro Plus 6.0 software. The average length of the 10 longest hair follicles in each group are presented as mean ± SEM. Group differences were obtained using the ANOVA test. The significance level for all tests was P<0.05.

### Statistics

Experiments were performed in triplicate. The data are presented as the means ± the standard errors of mean (SEM). Differences between two independent groups were compared with Student's t-test.

## Results

### Effects of Cryopreservation of the Whisker Hair Follicle Behavior in Gelfoam Histoculture

**Fig 2:**
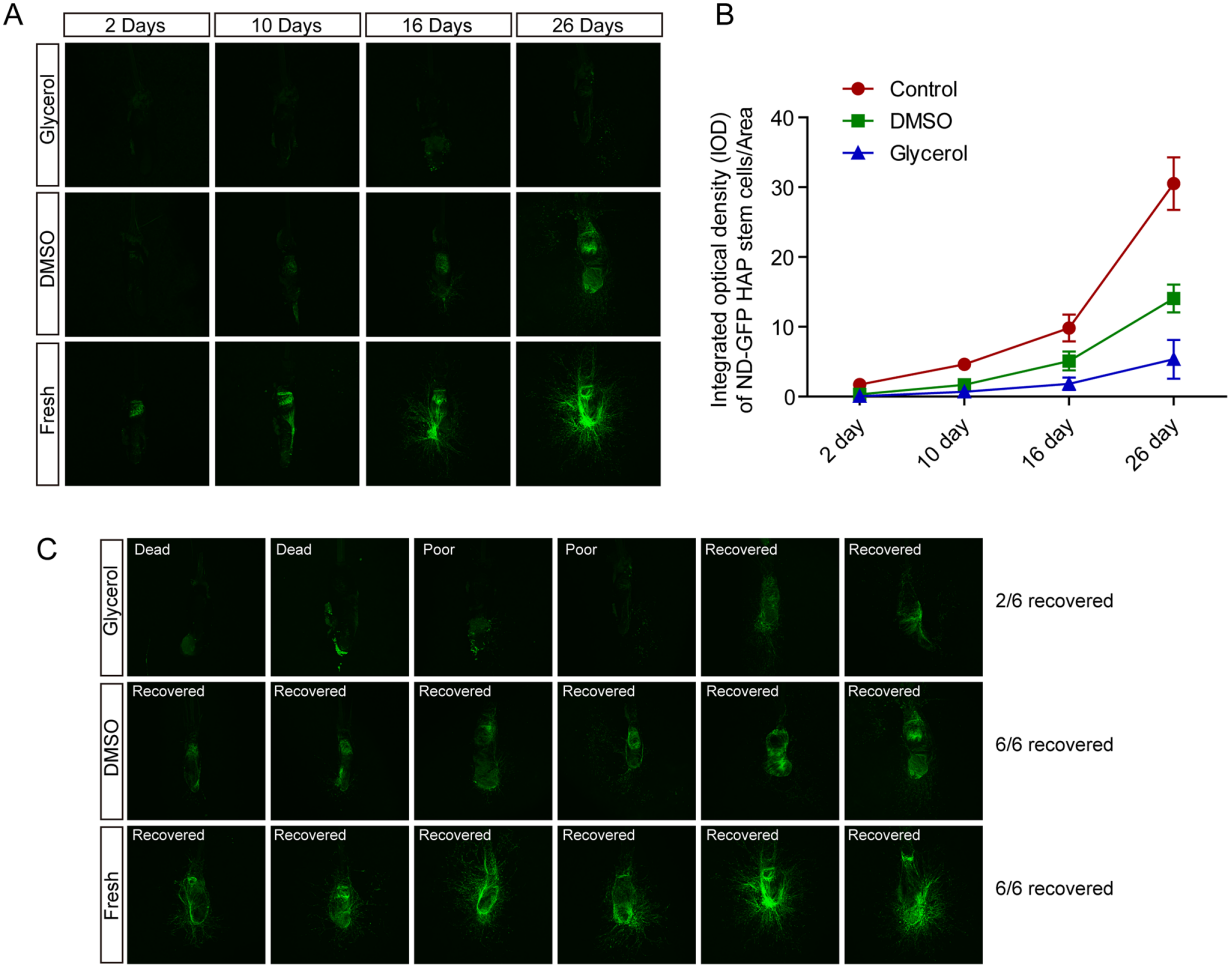
Confocal 3D images of ND-GFP hair-follicle-associated-pluripotent (HAP) stem cells in whisker follicles grown in Gelfoam histoculture. (**A**) ND-GFP-expressing HAP stem cells in DMSO- or glycerol-cryopreserved hair follicles or fresh follicles at day 2, 10, 16 and 26 days after Gelfoam histoculture. (**B**) Quantitative analysis of ND-GFP HAP stem cells fluorescence in cultured whisker follicles. DMSO-cryopserved follicles had more extensive growth of HAP stem cells than in glycerol-cryopreserved follicles, but less than in fresh follicles. (**C**) At day 26 after culture on Gelfoam, only 2/6 hair follicles that were cryopreserved in glycerol recovered. In contrast, 6/6 hair follicles recovered after DMSO cryopreservation, similar to fresh follicles.

After thawing and culture on Gelfoam for 4 weeks, the ND-GFP fluorescence of the HAP stem cells was measured. Confocal real-time 3D images of ND-GFP HAP hair follicle on Gelfoam showed that ND-GFP HAP stem-cell-fluorescence in fresh hair follicle had dramatic increases at 10, 16 and 26 days. The increases in HAP stem cell ND-GFP fluorescence after DMSO cryopreservation of hair follicles was slower than in fresh follicles. Glycerol-cryopreserved follicles had less increases in ND-GFP fluorescence than the DMSO-preserved follicles (Fig 2A and 2B). At day 26, 2/6 hair follicles cryopreserved in glycerol recovered to grow ND-GFP HAP stem cells, 2/6 had poor growth, and 2/6 were consider dead. In contrast, 6/6 hair follicles frozen in DMSO recovered (Fig 2C).

**Fig 3:**
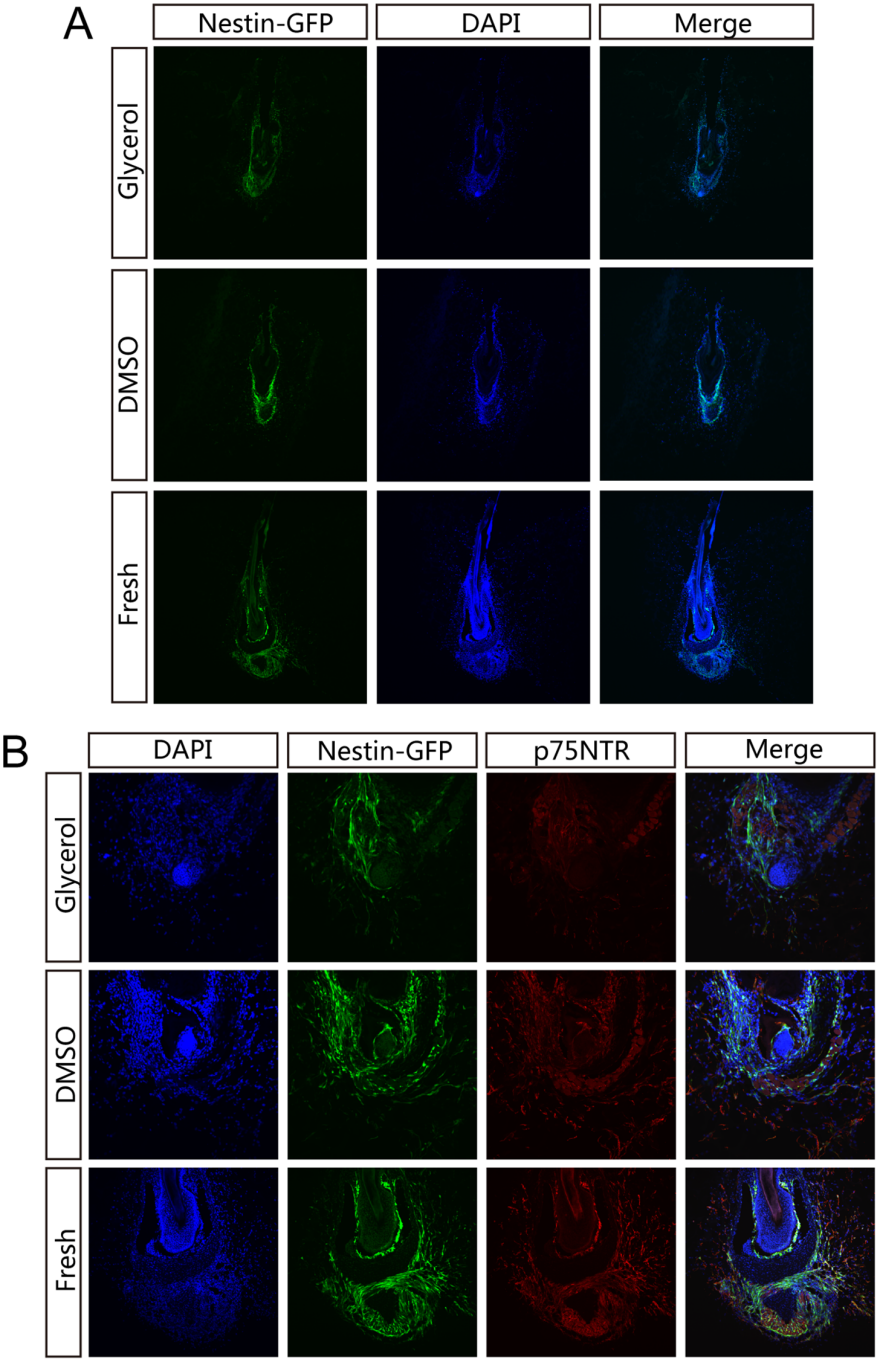
DAPI nuclear staining and P75^NTR^ immuostaining of cryopreserved or fresh follicles after 26 days in Gelfoam histoculture. (**A**) DAPI nuclear staining. Fresh hair follicles maintained intact structures of the hair shaft, inner root sheath, and outer root sheath, with a large amount of nestin-GFP HAP stem cells growing under the bulb area after 26 days of Gelfoam histoculture. In contrast, the inner structures of Gelfoam-histocultured hair follicles after DMSO- or glycerol-cryopreservation had no DAPI-stained nuclei, indicating damage of the inner structure of the follicle after cryopreservation. Most of the nestin-GFP expressing HAP stem cells grew around the outer root sheath and bulb area in cryopreserved hair follicles. (**B**) p75^NTR^ immunostaining of frozen sections colocalized with ND-GFP HAP stem cells. These results indicate that ND-GFP HAP stem cells in cryopreserved follicles were maintained.

After 26 days in Gelfoam histoculture, 3D confocal images showed that ND-GFP HAP stem cell outgrowth occurred both at the upper and lower part of fresh hair follicles (Fig 2C). DAPI nuclear staining showed that the fresh hair follicles maintained normal structures in the hair shaft, inner root sheath, and outer root sheath, and a large amount of ND-GFP HAP stem cells grew around the bulb area (Fig 3A). In DMSO cryopreserved follicles, ND-GFP 3D images indicated similar HAP stem cells outgrowth both in the upper and lower part of hair follicle, but with less cell outgrowth than in fresh hair follicles (Fig 2C). With glycerol-cryopreservation, the recovered hair follicles had less ND-GFP HAP stem cells. The cultured hair follicles had ND-GFP HAP stem cells mostly in the outer root sheath and the bulb area after both DMSO- and glycerol-cryopreservation. In contrast, the inner structure of cultured hair follicles had little DAPI nuclear staining remained after either method of cryopreservation. These results indicate cell death of the inner part and structural damage of the frozen hair follicle in both cryopreservation media (Fig 3A). The bulb area of the hair follicle had ND-GFP-nestin HAP stem cells growing out, and the dermal papilla area maintained DAPI nuclear staining after both cryopreservation methods. These results suggest the survival of bulb area and dermal papilla cells in both DMSO and glycerol-cryopreserved hair follicles (Fig 3B). p75^NTR^ immunostaining also indicated ND-GFP HAP stem cells were maintained in both conditions of cryopreservation (Fig 3B).

### Functional Recovery of Cryopreserved Hair Follicles after Subcutaneous Transplantation

After the hair follicles were cryopreserved in DMSO for one week at -80°C, we thawed the hair follicles and transplanted them subcutaneously in nude mice. Each flank was transplanted with 5–6 hair follicles and a total of 3 mice were used. Eight of 15 transplanted cryopreserved hair follicles grew out extensive long hair shafts.

**Fig 4:**
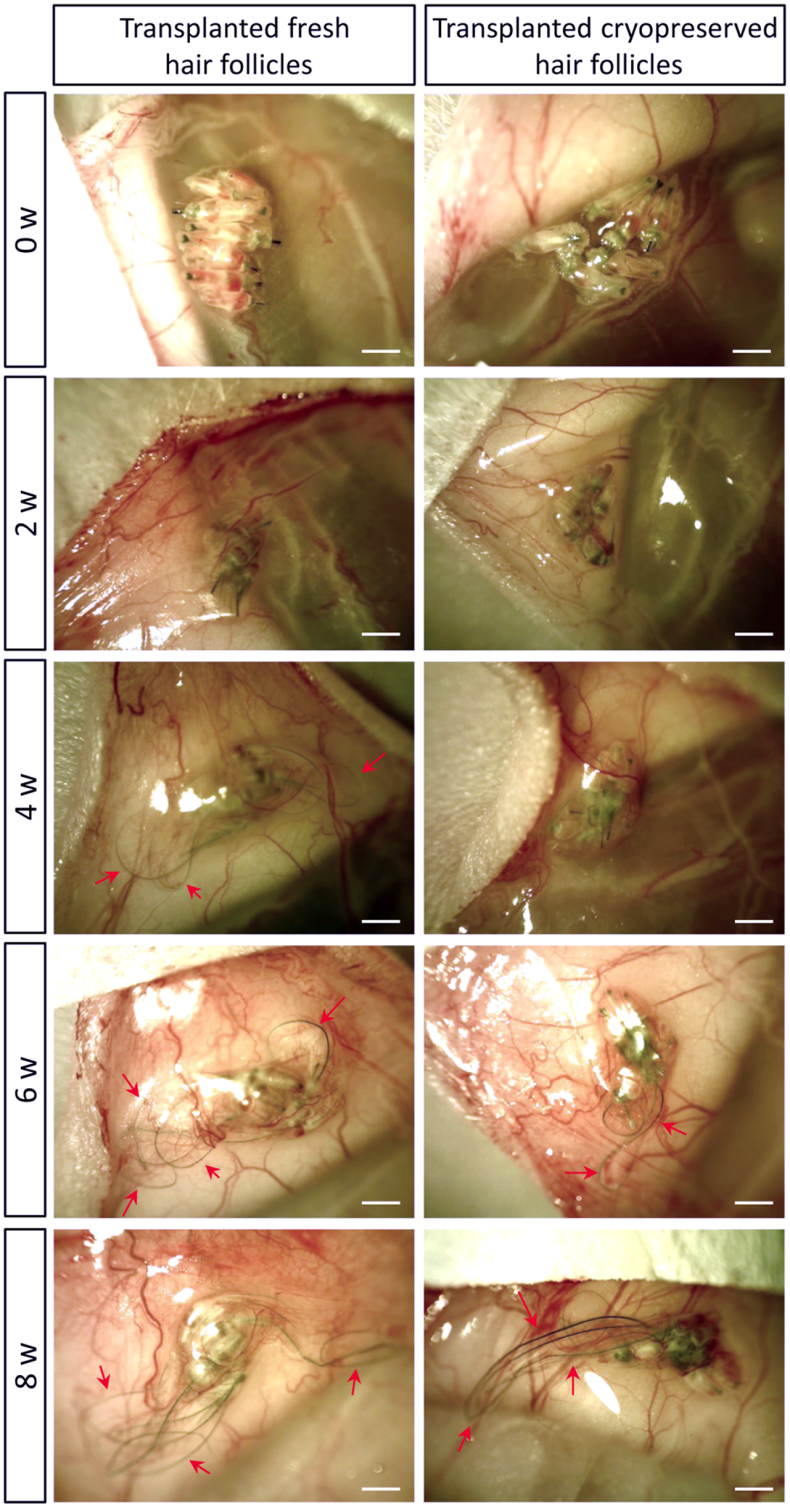
In vivo images comparing hair-shaft growth in fresh follicles and in DMSO cryopreservated follicles after subcutaneous transplantation. At 2 weeks after transplantation, fresh and DSMO-cryopreserved hair follicles established blood-vessel connections with the host nude mice, indicated by red blood in the lower and upper parts of the hair follicle. At week 4, there were more blood vessels surrounding the hair follicles. There were obvious hair roots in the bulb area and longer hair shafts at 4 weeks compared to 2 weeks, indicating the cryopreserved hair follicles started to grow hair shafts compared to fresh follicles. At 6 weeks, more hair follicles had hair roots in the bulb area and grew out long hair shafts in the transplanted fresh and cryopreserved hair follicles. At 8 weeks, there was a large number of long hair shafts. Bars = 1 mm.

**Fig 5:**
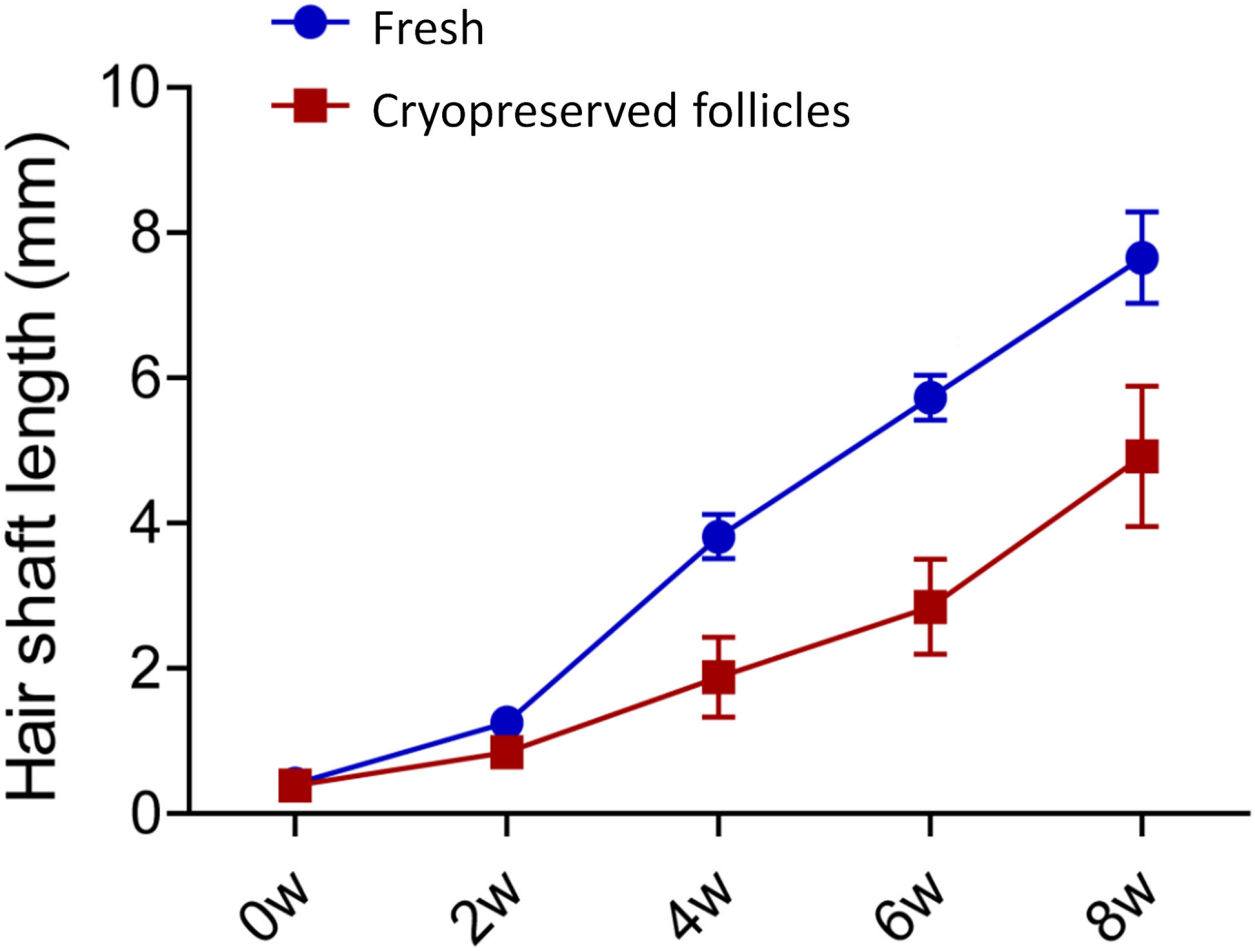
Quantitative data of hair shaft growth in fresh and DMSO-cryopreserved hair follicles after hair follicle subcutaneous transplantation in nude mice. Each data point represents the 10 longest hair follicles for each group.

In fresh hair follicles, the hair follicle established blood vessel connection with the host nude mice and grew rapidly by week 2 (1.26 mm ± 0.15mm). At week 4, the average of the 10 longest hair shafts length was 3.82 mm ± 0.31 mm. At week 6, the average of the 10 longest hair shafts length was 5.73 mm ± 0.31 mm), and at week 8, the hair shafts length was 7.66 mm ± 0.63 mm. The DSMO-cryopreserved hair follicle also established blood vessel connections with the host nude mice as indicated by red blood in the lower part and upper part of the hair follicle. At week 4, there were more blood vessels surrounding the hair follicles. There were obvious hair roots at the bulb area as well as longer hair shafts shaft at week 4, with the longest length of 1.88 mm ± 0.55 mm, compared to week 2, when the longest hair shaft length was 0.85 mm ± 0.07 mm (Fig 4). These results indicated that the DSMO-cryopreserved hair follicle started to rapidly regrow hair. At week 6, the longest hair shafts grew up to 2.85 mm ± 0.66 mm and more hair roots in the bulb area were observed, as well as long hair shafts growing out. Eventually, at week 8, there were a large amount of long hair shafts with the longest at a length of 4.92 mm ± 0.97 mm (Fig 5). Our results showed that DSMO-cryopreserved hair follicles maintained the ability to produce hair shafts after subcutaneous transplantation.

## Discussion

We have described a general cryopreservation method to preserve the hair follicle, and it’s functional recovery of producing hair shafts after thawing and subcutaneous transplantation. At first, we compared two commonly used cryopreservation reagents, glycerol and DMSO, to maintain the hair follicle. DMSO preserved the hair follicle with a higher recovery rate of large populations of ND-GFP HAP stem cells than glycerol, as determined in Gelfoam histoculture. DSMO-preserved HAP stems cells were also indicated by the presence of the neural crest stem-cell marker P75^NTR,^. The functional recovery of the cryopreserved hair follicle was further demonstrated by extensive hair-fiber growth over 8 weeks, after subcutaneous transplantation to nude mice.

The cryopreserved hair follicles established blood vessel connections with host nude mice, which could provide nutrients and oxygen to the hair follicle stem cells, enabling hair shaft growth. Two weeks after transplantation, the cryopreserved hair follicle started to produce growing hair shafts, indicating functional recovery. Hair shaft production occurred after in vivo transplantation of cropreserved hair follicles, even though in parallel experiments of culture of cryopreserved hair follicles on Gelfoam for 26 days, frozen-section staining indicated damaged structures inside the follicle. Therefore, the fact that the cryopreserved hair follicles produced hair shafts after subcutaneous transplantation suggests a powerful supporting system in vivo. Our results also showed that ND-GFP HAP stem cell in the in bulb area and dermal papilla after cryopreservation. Previous reports showed the dermal papilla is of prime importance in the growth of hair [13] and dermal stem cells are involved in hair growth [14]. Therefore, the bulb area cells may be mainly responsible for hair shaft growth after in vivo transplantation and recovery.

The present manuscript demonstrates the proof-of-principle that whisker follicles could be cryopreserved such that they could growth again after thawing and transplanted in nude mice. This is an important step toward general cryopreservation of hair follicles. Time course cryopreservation will be done in future experiments.

The whisker is a giant hair follicle and ideal for hair research. The successful cryopreservation results with the whisker in the present report suggest that small follicles, such as pelage, would be readily cryopreserved as well, especially since small specimens are generally easier to cryopreserve. Future experiments will address this issue.

The functional recovery of frozen hair follicles after subcutaneous transplantation suggests the use of hair-follicle cryo-banks for transplantation for later hair loss problems, for example, alopecia. The thawed follicles could also be used for transplantation to regenerate injured peripheral nerves [4, 15–17] or the spinal cord [5, 18].

## Dedication

This paper is dedicated to the memory of A. R. Moossa, M.D.

## Supporting Information

S1 ARRIVE Checklist (PDF)
